# On the Diuretic Effects of Mercury in a Case of Syphilis

**Published:** 1807-10

**Authors:** James S. Stringham

**Affiliations:** Professor of Chemistry in Columbia College, *N. America*


### *On the diuretic Effects of Mercury in a Case of Syphilis.*

*By* James S. Stringham, M. D. Professor of Chemistry in Columbia College, *N. America*.

The subject of the following case, Mr. A —— B ——, applied to me early in the month of October last, in consequence of a small ulcer in the throat, which he suspected to be the effect of a syphilitic taint, as he had had chancres during the summer before. It appeared that for the cure of these he had taken a very considerable quantity of mercury, though his mouth had been but slightly affected by it. I advised him again to adopt a mercurial course; and for this purpose furnished him with the pilulæ hydrargyri of the Ed. Pharm. Of these he began with one, morning and evening, but finding no inconvenience of the stomach stomach or bowels from them, he gradually increased the quantity to eight each day. These, together with the decoct. lignor. were continued from the 30th of October to the 19th of December, when by my particular request he desisted from any further use of the remedy. I was much surprised to find that, notwithstanding the great number of pills which he had taken, and the great quantity of mercury which must necessarily have been introduced into his system, no perceptible effects had been produced either upon the salivary glands, the gums, or the breath. On inquiry, he informed me that he had sometimes experienced a slight soreness of the mouth while taking the pills, that this was soon succeeded by an unusual flow of urine, after which every trace of the mercury entirely disappeared. This presented to me a case of a complexion entirely novel, and I determined to make another effort to produce an effect which I considered as essential to his safety. Accordingly, he again commenced taking mercury combined with opium, and every precaution was taken to guard against its action as a diuretic—but in vain; no sooner was any part of the fauces affected by the medicine, than a præternatural secretion took place in the kidneys; and although he continued to take his pills regularly, his mouth became sound as though they had never been exhibited. With respect to his throat, the ulcer began to mend shortly after he commenced the mercurial course, and in about three weeks entirely disappeared; but it has since occasionally returned, though by the application of caustic it was removed in two or three days. I may remark, that during the exhibition of the mercury, neither the stomach nor bowels of my patient were in any way affected by it.

It is very possible that cases of a similar kind may have occurred in the private practice of other physicians, but I do not recollect that any instance has been recorded, where such a regular determination to the kidneys took place from the action of mercury, so effectually counteracting the objects for which it was intended. I confess that I feel no small degree of embarrassment with respect to that plan of treatment which will most effectually guard against the recurrence of syphilitic symptoms in this case, and more particularly as to what are the data, on which I may venture to pronounce my patient secured against any future inconvenience from his present infection.

I have heard physicians complain of the great difficulty they sometimes found in producing salivation. May not the same obstacle have existed in such instances as in the case case just related? And where too profuse a salivation supervenes, may we not infer from this case, that diuretics are the most effectual means by which to procure an alleviation of symptoms?

